# Process Optimization of Electrochemical Treatment of COD and Total Nitrogen Containing Wastewater

**DOI:** 10.3390/ijerph19020850

**Published:** 2022-01-13

**Authors:** Jiachao Yao, Yu Mei, Junhui Jiang, Guanghua Xia, Jun Chen

**Affiliations:** 1 College of Biology and Environmental Engineering, Zhejiang Shuren University, Hangzhou 310015, China; jcyao@zjut.edu.cn (J.Y.); imy1220@zjut.edu.cn (Y.M.); 2 The Engineering Technology Center of Pollution Control in Taizhou, Taizhou 318000, China; 14060601003@pop.zjgsu.edu.cn; 3 College of Life Science, Taizhou University, Taizhou 318000, China; zhouwuluyao@sjtu.edu.cn

**Keywords:** electrochemical oxidation, electrochemical reduction, COD, total nitrogen, wastewater treatment

## Abstract

In this work, an electrochemical method for chemical oxygen demand (COD) and total nitrogen (TN, including ammonia, nitrate, and nitrite) removal from wastewater using a divided electrolysis cell was developed, and its process optimization was investigated. This process could effectively relieve the common issue of NO_3_^−^/NO_2_^−^ over-reduction or NH_4_^+^ over-oxidation by combining cathodic NO_3_^−^/NO_2_^−^ reduction with anodic COD/NH_4_^+^ oxidation. The activity and selectivity performances toward pollutant removal of the electrode materials were investigated by electrochemical measurements and constant potential electrolysis, suggesting that Ti electrode exhibited the best NO_3_^−^/NO_2_^−^ reduction and N_2_ production efficiencies. In-situ Fourier transform infrared spectroscopy was used to study the in-situ electrochemical information of pollutants conversion on electrode surfaces and propose their reaction pathways. The effects of main operating parameters (i.e., initial pH value, Cl^−^ concentration, and current density) on the removal efficiencies of COD and TN were studied. Under optimal conditions, COD and TN removal efficiencies from simulated wastewater reached 92.7% and 82.0%, respectively. Additionally, reaction kinetics were investigated to describe the COD and TN removal. Results indicated that COD removal followed pseudo-first-order model; meanwhile, TN removal followed zero-order kinetics with a presence of NH_4_^+^ and then followed pseudo-first-order kinetics when NH_4_^+^ was completely removed. For actual pharmaceutical wastewater treatment, 79.1% COD and 87.0% TN were removed after 120 min electrolysis; and no NH_4_^+^ or NO_2_^−^ was detected.

## 1. Introduction

A large quantity of wastewater produced from industrial manufacturing process is contaminated by two often-regulated primary contaminants: organic matter and nutrients, such as nitrogen in the forms of ammonia, nitrate, and nitrite [1,2]. Biological technology has been considered an effective method for chemical oxygen demand (COD) and total nitrogen (TN) removal from wastewater [3,4], but rigorous monitoring (e.g., pH, temperature) is needed to maintain daily operations [5,6]. Several other methods, such as membrane filtration [7,8], ion exchange [9,10], and adsorption [11], are also extensively used to remove COD and nitrogen contamination; however, these techniques may result in secondary pollution [12,13].

Electrochemical method is proposed as a promising alternative for wastewater treatment because of its high efficiency, versatility, easy operation, and environmental compatibility [14,15,16]. Ganzoury et al. [17] summarized that, compared with traditional biological technology, the electrochemical method is much more suitable for removal of persistent color and pollutants from wastewater. In recent literatures, many scholars investigated the anodic oxidation of COD or NH_4_^+^ and proved the superiority of electrochemical wastewater treatment [18,19]. For example, Zöllig et al. [20] reported that, for real stored urine treatment by electrochemical method, ammonia could be efficiently removed by direct and indirect oxidation. In addition, many researches focusing on cathodic reduction of NO_3_^−^ or NO_2_^−^ have been reported [21,22]. Su et al. [13] presented the performances of Co_3_O_4_/Ti, Fe_2_O_3_/Ti, and Cu cathodes on NO_3_^−^ removal, and Co_3_O_4_/Ti exhibited better NO_3_^−^ removal efficiency.

Current studies for wastewater treatment by electrochemical method mainly concentrate on the removal of a single pollutant or two mixed pollutants, and there are few investigations on the electrochemical treatment of wastewater containing COD, NH_4_^+^, NO_3_^−^, and NO_2_^−^. In our previous work [23], the feasibly of simultaneous removal of COD and TN was proven; however, it should be noticed that one of the issues for COD and TN removal is to restrain the problems of side reactions of NH_4_^+^ over-oxidation on anode and NO_3_^−^/NO_2_^−^ over-reduction on cathode. Actually, many literatures indicate that the over-oxidation or over-reduction is a common phenomenon in the process of nitrogen-containing wastewater treatment by electrochemical method [24,25,26], which means that the TN removal is limited. For instance, Shih et al. [27] studied the electrochemical performance of ammonia oxidation over a modified nickel foam electrode. The results indicated that, though high ammonia removal rate was achieved, N-atom was easily over-oxidized to NO_3_^−^ and NO_2_^−^. Li at al. [28] investigated the NO_3_^−^ reduction with Fe cathode, and the concentration of NO_3_^−^ decreased from 100.0 to 7.2 mg-N·L^−1^ in 3 h, but 51.1 mg-N·L^−1^ NH_4_^+^ was generated as the by-product. In order to relieve the issue of over-reduction/oxidation, it is essential to find a suitable reaction cell and key operating parameters to perform the balance between cathodic reduction and anodic oxidation. Li et al. [29] and Mandal et al. [30] reported that a divided cell was more proper for electrochemical denitrification than undivided cell thanks to the ion-exchange membrane, which could reduce the interactions between anodic and cathodic reactions. Many investigators [31,32] also presented that operating parameters, e.g., electrode material, current density, NaCl concentration, pH, flow rate, and temperature, could strongly affect the electrochemical performance of pollutant removal.

In this work, an electrochemical process for COD and TN removal was optimized, and its feasibility to relieve the issue of over-oxidation or over-reduction was evaluated. Ti, Cu, and stainless steel were selected as cathode material to study their reduction performance for NO_3_^−^/NO_2_^−^ removal. The in-situ electrochemical information of COD/NH_4_^+^ oxidation and NO_3_^−^/NO_2_^−^ reduction on electrode surfaces were explored by in-situ Fourier transform infrared spectroscopy (in-situ FTIR). The key operating parameters, i.e., initial pH value, chloride concentration, and current density, were studied to perform cathodic reduction of NO_3_^−^/NO_2_^−^ and anodic oxidation of COD/NH_4_^+^. Additionally, kinetics and reaction mechanism were also proposed to describe the redox process. Finally, an actual pharmaceutical wastewater was investigated to verify the electrochemical performance for COD and TN removal.

## 2. Materials and Methods

### 2.1. Wastewater Characteristics

Simulated wastewater containing COD and TN was prepared by dissolving ethyl acetate, ammonium sulfate, sodium nitrate, and sodium nitrite into 0.1 mol L^−1^ Na_2_SO_4_ solution. Sodium chloride was added into the solution to maintain the concentration of chloride ion. Actual pharmaceutical wastewater, which was characterized by low biodegradability and complex composition, was collected from a pharmaceutical factory (Zhejiang, China). The wastewater characteristics are summarized in Table 1.

### 2.2. Experimental Setup

Electrochemical experiments were conducted in a divided electrolysis cell (Figure S1). Ti/PbO_2_ electrode (10 cm × 10 cm) was used as anode, while Ti, Cu, and stainless steel were selected as cathode, respectively. A volume of 600 mL wastewater was circulated between anode and cathode chamber by a peristaltic pump.

Electrochemical measurements (i.e., steady-state polarization curves and linear sweep voltammograms) and constant potential electrolysis were executed for the choice of a proper cathode with an electrochemical workstation. The different cathodes were used as the working electrode; platinum sheet and saturated calomel electrode (SCE) were employed as the counter and reference electrode, respectively. In-situ FTIR spectra were obtained by a FTIR spectrometer equipped with a self-made spectroelectrochemical cell (Figure S2).

### 2.3. Analytical Methods

COD, TN, and ammonia were determined by the dichromate, alkaline potassium persulfate, and Nessler reagent spectrophotometry, respectively [4,13]. Anions (e.g., nitrate, nitrite, and chloride) were determined by ion chromatography. Nitrogen oxides were monitored by gas chromatography. The pollutant removal efficiency, product selectivity, and energy consumption were calculated based on our previous work [33].

## 3. Results

### 3.1. The Choice of a Proper Electrode

In the electrochemical system for COD and TN removal, both anode and cathode materials are conclusive for optimizing the redox process [34]. The investigations of anode selection had been reported in our previous work: commercial electrode materials of Ti/PbO_2_, Ti/IrO_2_, Ti/RuO_2_, and BDD were compared for treatment of typical industrial wastewater, suggesting that Ti/PbO_2_ anode was suitable for COD and NH_4_^+^ removal [35,36]. Similarly, cathode material plays an important role in electrochemical denitrification. From the view of practical applications, Ti, Cu, and stainless steel were selected as cathode materials in this work to study the electrochemical properties and NO_3_^−^/NO_2_^−^ removal efficiencies due to their high stability and low cost.

**Figure 1:**
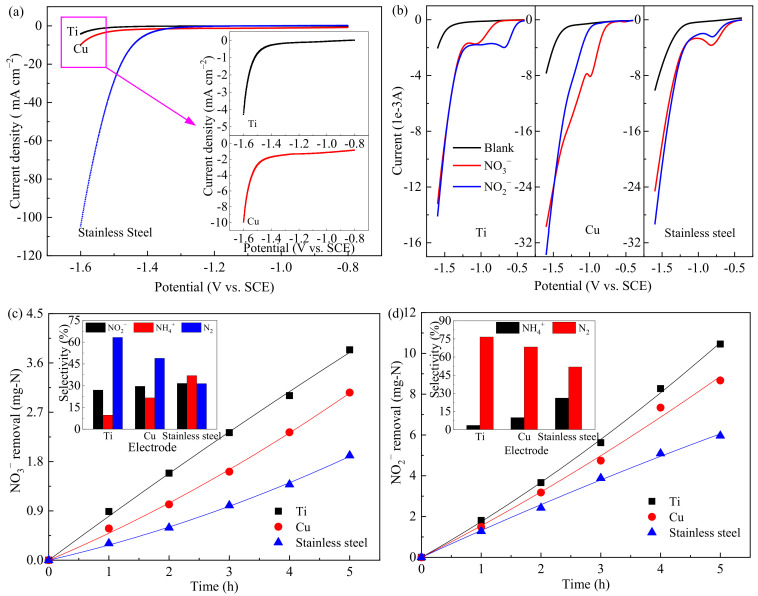
Electrode characterization and pollutant removal. (**a**) Steady-state polarization curves; (**b**) linear sweep voltammograms (blank: 0.1 mol·L^−1^ Na_2_SO_4_); (**c**) NO_3_^−^ reduction (0.1 mol L^−1^ Na_2_SO_4_ + NO_3_^−^ solution), and the inset shows the selectivity of nitrogen species after 5-h electrolysis; (**d**) NO_2_^−^ reduction (0.1 mol·L^−1^ Na_2_SO_4_ + NO_2_^−^ solution), and the inset shows the nitrogen species after 5-h electrolysis.

Figure 1a shows the steady-state polarization curves of the hydrogen evolution reactions using three cathodes at a scan rate of 10 mV·s^−1^. Results indicated that Ti electrode had a hydrogen evolution potential of −1.542 V, which was more negative than those of Cu (−1.532 V) and stainless steel (−1.466 V), meaning that a side reaction of hydrogen evolution was least likely to occur on Ti electrode. Figure 1b presents the linear sweep voltammograms of three electrodes on NO_3_^−^/NO_2_^−^ reduction. Obvious cathodic peaks and extended broad waves were observed with the addition of NO_3_^−^/NO_2_^−^ into the blank solution, which meant the direct NO_3_^−^/NO_2_^−^ reduction occurred. Cathodic peaks of Ti, Cu, and stainless steel were at about −1.10, −0.99, and −0.82 V for NO_3_^−^ reduction, respectively. For NO_2_^−^ reduction, cathodic peaks of −0.68 and −0.81 V were observed for Ti and stainless steel (not observed on Cu), respectively.

The removal and selectivity of NO_3_^−^/NO_2_^−^ reduction with three electrodes at a constant potential of −1.26 V are shown in Figure 1c,d. Figure 1c displays that the highest electrocatalytic performance for NO_3_^−^ reduction was obtained with the Ti cathode, while that with stainless steel was the lowest. The inset of Figure 1c indicates that NO_2_^−^ and NH_4_^+^ were the intermediates or by-products during the NO_3_^−^ reduction process [37,38]. The order of N_2_ selectivity of these three electrodes was Ti (63.3%) > Cu (48.9%) > stainless steel (31.5%). This result might be related to the relative hydrogen evolution potential shown in Figure 1a. Su et al. [13] and Reyter et al. [39] presented that NH_4_^+^ as the NO_3_^−^ reduction by-product was favored in a potential region closing to the hydrogen evolution reaction region; in other words, more negative hydrogen evolution potential led to higher N_2_ selectivity. The information regarding NO_2_^−^ reduction from wastewater with different cathodes as working electrode is shown in Figure 1d. The amounts of NO_2_^−^ reduction for Ti, Cu, and stainless steel were 10.46, 8.67, and 5.96 mg-N in 5 h; the N_2_ selectivities with these three electrodes were 76.6%, 68.4%, and 51.8%, respectively. According to the results shown in Figure 1, the electrochemical properties and catalytic activities of the three cathodes for NO_3_^−^/NO_2_^−^ reduction decreased in the following order: Ti > Cu > stainless steel; the Ti electrode was therefore selected for subsequent experiments to study the optimal reaction conditions.

### 3.2. In-Situ FTIR Studies on Pollutants Removal

**Figure 2:**
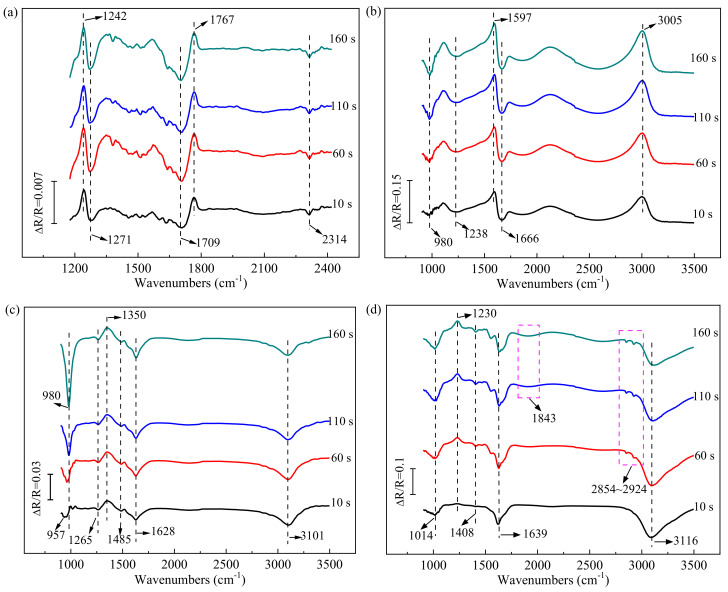
In−situ FTIR spectra collected in electrolysis of COD (**a**), NH_4_^+^ (**b**), NO_3_^−^ (**c**), and NO_2_^−^ (**d**) solution with electrolysis time.

To explore solid/liquid interfacial phenomena, i.e., identify the intermediates, adsorbed species, and products during pollutants removal, in-situ FTIR spectroscopy was applied in the studies of oxidation and reduction processes (Figure 2). In the spectrum, a positive band represents the consumption of compound, and a negative band represents the generation of product.

As shown in Figure 2a, the ester bond of the organic is confirmed at the positive bands of 1242 and 1767 cm^−1^ during COD electro-oxidation. Two negative infrared (IR) bands are observed at about 1271 and 1709 cm^−1^, representing the C−OH and C=O scissors vibration in COOH, respectively. The downward band at 2314 cm^−1^ is assigned to CO_2_ absorption. The results indicated that the organic matter was decomposed to low molecule acid and then converted to CO_2_.

Figure 2b shows that the vibrations of NH_4_^+^ are detected at 1597 and 3005 cm^−1^. The weak bands located at 980 and 1666 cm^−1^ represent the absorption of NH_3,ads_. Besides, dispersed bands are observed around 2000–3000 cm^−1^. According to the study by Griffiths and Haseth [40], the complex bands may be attributed to the vibrations of NH_3_^+^, NH_2_^+^, and NH^+^. A band at 1238 cm^−1^ can be also observed, indicating the generation of NO_2_^−^ [40]. Though NO_2_^−^ band was observed, the vibration of NO_3_^−^ was not shown. This phenomenon might be related to the operating conditions. For example, Kapałka et al. [41] indicated that ammonia removal and product distribution were strongly pH dependent. Likewise, operating parameters, e.g., chloride concentration and current density, could also affect the distribution of products during NH_4_^+^ electro-oxidation because of the concentrations of generated active radicals, which was reported by Anglada et al. [42]. On the basis of experimental results, a simple reaction route of NH_4_^+^ electro-oxidation in this system was proposed as shown in Equation (1):

(1) $$\begin{array}{ll} NH_{4}^{+}\;↔\;NH_{3,ads}\;\to \;NH_{3}^{+}\;\to \;NH_{2}^{+}\;\to & NH^{+}\;\to \;N_{2}\\ & ↓\\ & NO_{2}^{-} \end{array}$$

The in-situ FTIR spectra measured during electro-reduction of NO_3_^−^ are given in Figure 2c. The IR absorption at 1350 cm^−1^ is ascribed to the existence of NO_3_^−^. The peak of 1265 cm^−1^ suggests the presence of NO_2_^−^ in electrolysis. Dispersed bands are presented at ca. 1800–3000 cm^−1^, indicating the vibrations of NH_2_^+^ and NH_3_^+^ [43]. Three characteristic IR bands at 957–980, 1628, and 3101cm^−1^ are observed and assigned to absorbed NH_3,ads_, and the weak band at 1485 cm^−1^ is confirmed as NH_4_^+^. As shown in Figure 2d, the band for NO_2_^−^ appears at 1230 cm^−1^. Similar to the spectra of NO_3_^−^, NH_3,ads_ generated at the bands of 1014, 1639, and 3116 cm^−1^ in the electro-reduction of NO_2_^−^, and then, the NH_4_^+^ is detected at 1408 cm^−1^. Additionally, weak bands at about 1843 and 2854–2924 cm^−1^ are discovered with electrolysis time, which are ascribed to volatile by-products of NO and NO_2_, respectively [44]. Similar to the condition of NH_4_^+^ oxidation, nitrate/nitrite reduction and products distribution also highly rely on operating parameter, such as optimum voltage, electrode distance, and initial pH [45]. In this electrochemical system, the NO_3_^−^/NO_2_^−^ electro-reduction mechanism was speculated in Equation (2), according to the FTIR spectra.

(2) $$\begin{array}{lll} NO_{3}^{-}\;\to & NO_{2}^{-}\;\to & \;NH_{2}^{+}\;\to \;NH_{3}^{+}\;\to \;NH_{3,ads}\;↔\;NH_{4}^{+}\\ & ↓\;↓ & \\ & N_{2}\;NO\;\to \;NO_{2} & \end{array}$$

### 3.3. Main Factors on COD and TN Removal

As stated above, operating parameters can make great influences on the performance of electrochemical process; moreover, actual wastewater is known for its characteristics of complexity and variety. Therefore, main factors, including initial pH value, chloride concentration, and current density, were investigated on COD and TN removal.

#### 3.3.1. Effect of Initial pH Value

**Figure 3:**
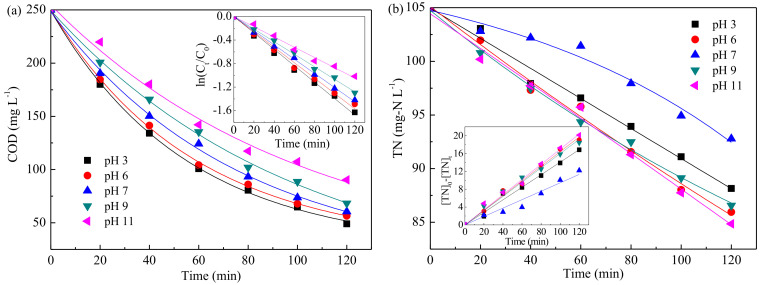
Effect of initial pH on COD (**a**) and TN (**b**) removal (no Cl^−^, 10.0 mA·cm^−2^, diluted H2SO4, and NaOH solutions were used for pH adjustments, and pH 6 was the natural pH of simulated wastewater).

Figure 3 presents the variations of COD and TN removal as a function of initial pH value. The COD removal efficiency decreased with the increase of pH value. However, the TN removal increased with the increase of pH value in acidic and non-acidic conditions, respectively. The inset of Figure 3a presents that a linear relationship was observed between the logarithm of COD concentration and electrolysis time; i.e., pseudo-first-order kinetics model emerged. Table S1 displays the apparent reaction rate constants K. As shown, the value of K at pH 3 was about 1.6 times that of pH 11, suggesting that COD removal was more favorable in acidic solution, which was in agreement with the findings by Chen et al. [32] and Zhao et al. [46]. Figure 3b shows that zero-order kinetics provided a suitable description of TN removal, and the rate constants K’ were listed in Table S1. The value of K’ increased about 1.2 times as pH from 3 to 6 and 1.8 times as pH from 7 to 11; however, the lowest rate was found at pH 7. Besides, it was observed that the TN removal efficiencies were close at pH values of 6, 9, and 11. This phenomenon might be associated with the effects of pH value on NO_3_^−^, NO_2_^−^, and NH_4_^+^ removals. As shown in Figure S3, acidic condition was more favorable for NO_3_^−^ and NO_2_^−^ reduction than neutral or alkaline condition due to the H-atom adsorbed on the surface of electrode, which was necessary for the NO_3_^−^/NO_2_^−^ indirect reduction [47]. However, a small quantity of NO was detected as by-product (Equation (3)) under the strong acid condition (pH ≤ 3) [48]. Moreover, Figure S3 displays that the removal efficiency of NH_4_^+^ was negative, suggesting that more NH_4_^+^ was generated in electrolysis rather than being removed. This could be explained by the over-reduction of NO_3_^−^/NO_2_^−^; i.e., NH_4_^+^ was generated as by-product in the process, indicating that the side reaction of over-reduction was the fundamental reason for limiting the TN removal in this electrochemical system. Taking into account the above results, initial pH value of 6 was selected for the further experiments.

(3) $$3NO_{2}^{-}+NO_{3}^{-}+6e^{-}+10H^{+}\to 5H_{2}O+4NO_{\left(g\right)}$$

#### 3.3.2. Effect of Chloride Concentration

**Figure 4:**
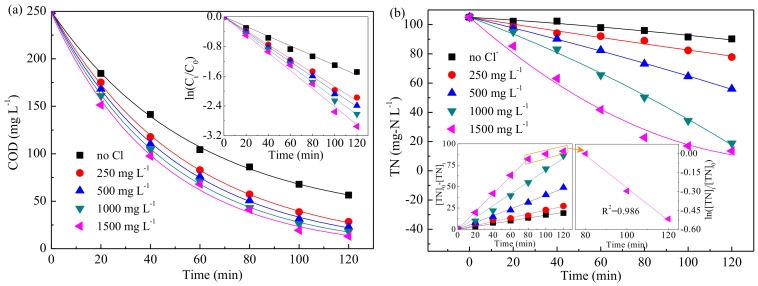
Influence of Cl^−^ concentration on COD (**a**) and TN (**b**) removal. (pH = 6, 10.0 mA·cm^−2^).

Figure 4 shows the simultaneous removal of COD and TN with the Cl^−^ concentration varied from 0 to 1500 mg·L^−1^. The results indicated that, as Cl^−^ was added, the removal of COD improved from 77.4% (0 mg·L^−1^·Cl^−^) to 88.6% (250 mg·L^−1^·Cl^−^), then increased slightly when further increasing Cl^−^ concentration. The TN removal efficiency increased from 18.1% to 26.0%, 46.7%, 82.0%, and 87.0% with the increase of Cl^−^ from 0 mg·L^−1^ to 250, 500, 1000, and 1500 mg·L^−1^, respectively. Besides, COD removal at different Cl^−^ concentrations followed pseudo-first-order kinetics. Meanwhile, the process of TN removal matched a zero-order model as NH_4_^+^ existed and then fitted on pseudo-first-order kinetics as NH_4_^+^ removed completely. According to the removal efficiency and rate constant, it seemed that Cl^−^ concentration of 1000 mg·L^−1^ was suitable for the next investigations.

#### 3.3.3. Effect of Current Density

**Figure 5:**
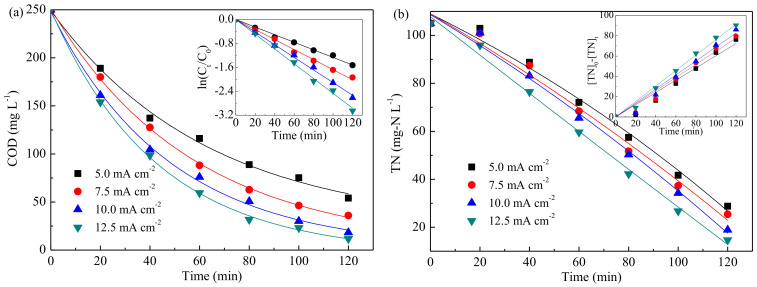
Effect of current density on COD (**a**) and TN (**b**) removal. (pH = 6, 1000 mg L^−1^ Cl^−^).

Figure 5 shows that increase of current density was beneficial to enhance the COD and TN removal efficiencies. The COD removal efficiency increased from 78.4% to 95.3% as current density increased from 5.0 to 12.5 mA cm^−2^; meanwhile, the TN removal efficiency increased from 72.6% to 86.0%. Figure 5 also displays that the pseudo-first-order and zero-order kinetics were still suitable for describing the COD and TN removal, respectively. As shown in Table S1, when current density improved from 5.0 to 12.5 mA·cm^−2^, the corresponding rate constant for COD removal increased from 1.269 × 10^−2^ to 2.471 × 10^−2^ min^−1^; and rate constant for TN raised from 6.032 to 7.918 mg·L^−1^·min^−1^. Though increasing current density was conducive to pollutant removal, the energy consumption would increase obviously. Current density of 10 mA·cm^−2^ was thus selected as the optimal one.

### 3.4. Verification of Actual Wastewater Treatment

**Figure 6:**
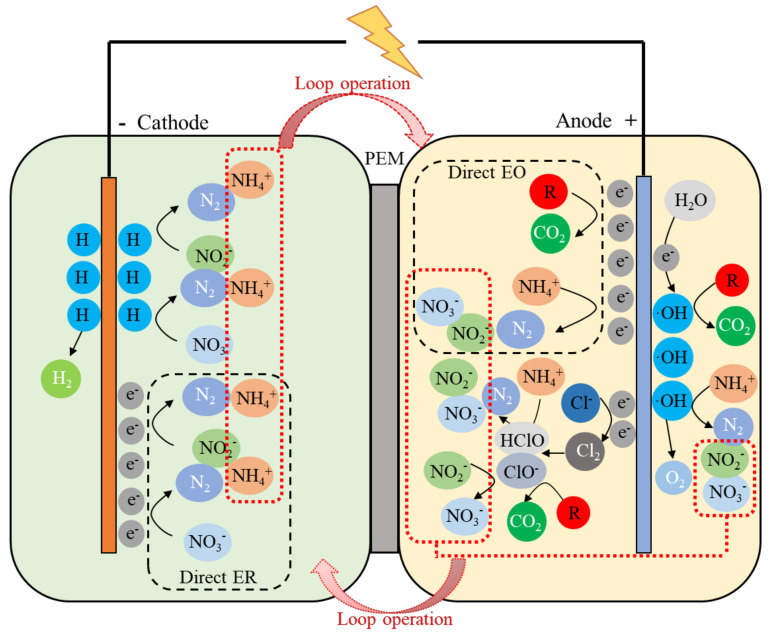
Schematic diagram of the electrolysis system. ER, electrochemical reduction; EO, electrochemical oxidation; R, organic compounds; PEM, proton−exchange membrane.

Considering to literatures and experimental results, a mechanistic model of electrochemical treatment of COD and TN containing wastewater in a divided electrolysis cell is proposed in Figure 6 [4,24]. In the process, the by-product of NH_4_^+^ generated during NO_3_^−^/NO_2_^−^ reduction was circulated into anode chamber by loop operation and then oxidized to N_2_ on anode; meanwhile, the NO_3_^−^/NO_2_^−^ as the over-oxidation by-product of NH_4_^+^ could be recirculated into the cathode chamber and continuously reduced to N_2_. This process could relieve the common issues of over-reduction and over-oxidation by combining cathodic NO_3_^−^/NO_2_^−^ reduction with anodic COD/NH_4_^+^ oxidation and thus improve the TN removal.

**Figure 7:**
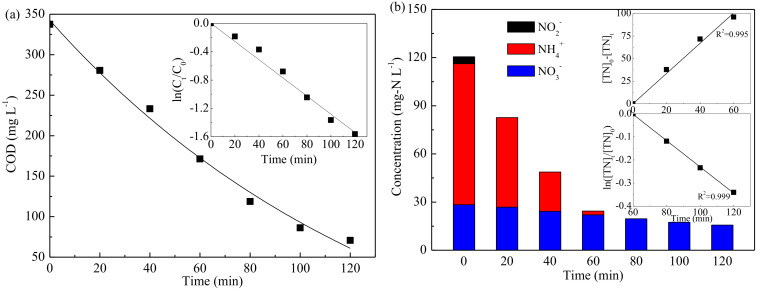
The simultaneous removal of COD (**a**) and total nitrogen (**b**) in actual pharmaceutical wastewater treatment.

To further investigate the feasibility for COD and TN removal by this electrochemical method, an actual pharmaceutical wastewater was sampled and treated in this study. Figure 7 shows the variations of COD and TN removal with electrolysis time. As shown, COD was effectively reduced from 337.57 to 70.64 mg·L^−1^ with the removal efficiency of 79.1%. Moreover, similar to the simulated wastewater treatment, COD removal in actual wastewater treatment followed pseudo-first-order kinetics. Figure 7b shows that the TN removal efficiency reached 87.0%; and no NH_4_^+^ or NO_2_^−^ was detected after 120 min treatment, while NO_3_^−^ was removed from 28.33 to 15.69 mg·L^−1^. The concentrations of nitrogen oxides were under the detection limit. In addition, Figure 7b shows that, with the presence of NH_4_^+^, TN removal followed zero-order kinetics model; however, when NH_4_^+^ was completely removed, pseudo-first-order kinetics was fitted. After 120 min electrolysis, the dissolved concentration of Pb^2+^ was measured to evaluate the safety of the treated wastewater; it was found that Pb^2+^ concentration was 0.008 mg/L, which met the Standard for Drinking Water Quality in China (plumbum ≤ 0.01 mg/L). Beside, with removal of 79.1% COD and 87.0% TN, the energy consumption was calculated as 13.3 kWh·m^−3^, which was much lower than that of traditional electrochemical method reported by other authors (Table S2). Meanwhile, compared with biological method, the electrochemical process in this work exhibited remarkable superiorities, such as high efficiency, short treatment time, and easy operation [49,50].

## 4. Conclusions

This work optimized the electrochemical treatment of COD and TN containing wastewater with a divided cell. The effects of cathode material, initial pH value, Cl^−^ concentration, and current density were investigated on the COD and TN removal. At an optimized pH 6, Cl^−^ concentration of 1000 mg·L^−1^, and current density of 10 mA·cm^−2^, this method could remove 92.7% COD and 82.0% TN in simulated wastewater using Ti cathode and Ti/PbO_2_ anode. Besides, kinetics and reaction mechanism were also proposed to describe the electrochemical process of COD and TN removal. For treatment of an actual pharmaceutical wastewater, the extents of COD and TN removal were 79.1% and 87.0%, respectively.

## Figures and Tables

**Table 1:** The main characteristics of the applied wastewater.

Parameters	Simulated Wastewater	Actual Wastewater
pH	6.0 ± 0.1	8.1 ± 0.2
COD/mg·L^−1^	250 ± 15	337.57 ± 20
Total nitrogen (TN)/mg-N·L^−1^	105 ± 10	120.53 ± 15
Ammonia (NH_4_^+^/NH_3_)/mg-N·L^−1^	60 ± 5	87.88 ± 8
Nitrate (NO_3_^−^)/mg-N·L^−1^	30 ± 3	28.33 ± 5
Nitrite (NO_2_^−^)/mg-N·L^−1^	15 ± 2	4.32 ± 3
Chloride (Cl^−^)/mg·L^−1^	0~1500	987.95 ± 35

## Data Availability

All data are fully available without restriction. All relevant data are within the manuscript and its Supporting Information files.

## Supplementary Materials

The following are available online at https://www.mdpi.com/article/10.3390/ijerph19020850/s1: Table S1, The efficiencies for COD and TN removal at different initial pH values, Cl^−^ concentrations, and current densities; Table S2, Selected results reported on energy consumption of actual wastewater treatment by electrochemical method; Figure S1, Schematic diagram of the divided electrolysis cell; Figure S2, Schematic diagram of in-situ FTIR; Figure S3, Influence of initial pH on NO_3_^−^, NO_2_^−^, and NH_4_^+^ variations.

## References

[1] Tanikawa D., Kataoka T., Ueno T., Minami T., Motokawa D., Itoiri Y., Kimura Z. (2021). Seeding the drainage canal of a wastewater treatment system for the natural rubber industry with rubber for the enhanced removal of organic matter and nitrogen. Chemosphere.

[2] Munavalli G.R., Sonavane P.G., Koli M.M., Dhamangaokar B.S. (2022). Field-scale decentralized domestic wastewater treatment system: Effect of dynamic loading conditions on the removal of organic carbon and nitrogen. J. Environ. Manag..

[3] Pourbavarsad M.S., Jalalieh B.J., Harkins C., Sevanthi R., Jackson W.A. (2021). Nitrogen oxidation and carbon removal from high strength nitrogen habitation wastewater with nitrification in membrane aerated biological reactors. J. Environ. Chem. Eng..

[4] Ding J., Zhao Q.L., Zhang Y.S., Wei L.L., Li W., Wang K. (2015). The eAND process: Enabling simultaneous nitrogen-removal and disinfection for WWTP effluent. Water Res..

[5] Nguyen H.D., Babel S. (2022). Insights on microbial fuel cells for sustainable biological nitrogen removal from wastewater: A review. Environ. Res..

[6] Falås P., Wick A., Castronovo S., Habermacher J., Ternes T.A., Joss A. (2016). Tracing the limits of organic micropollutant removal in biological wastewater treatment. Water Res..

[7] Noriega-Hevia G., Serralta J., Secob A., Ferrer J. (2021). Economic analysis of the scale-up and implantation of a hollow fibre membrane contactor plant for nitrogen recovery in a full-scale wastewater treatment plant. Sep. Purif. Technol..

[8] Kimura K., Yamakawa M., Hafuka A. (2021). Direct membrane filtration (DMF) for recovery of organic matter in municipal wastewater using small amounts of chemicals and energy. Chemosphere.

[9] Tong S., Zhang S.X., Zhao Y., Feng C.P., Hu W.W., Chen N. (2022). Hybrid zeolite-based ion-exchange and sulfur oxidizing denitrification for advanced slaughterhouse wastewater treatment. J. Environ. Sci..

[10] Vincenta T.T., Boyer T.H. (2020). Beneficial reuse of treated municipal wastewater and flue gas carbon dioxide via combined ion exchange. J. Water Process Eng..

[11] Xiao W., Jiang X.P., Liu X., Zhou W.M., Garba Z.N., Lawan I., Wang L.W., Yuan Z.H. (2021). Adsorption of organic dyes from wastewater by metal-doped porous carbon materials. J. Clean. Prod..

[12] Li M., Feng C.P., Zhang Z.Y., Yang Y.N., Chen R.Z., Sugiura N. (2009). Simultaneous reduction of nitrate and oxidation of by-products using electrochemical method. J. Hazard. Mater..

[13] Su L.H., Li K., Zhang H.B., Fan M.H., Ying D.W., Sun T.H., Wang Y.L., Jia J.P. (2017). Electrochemical nitrate reduction by using a noval Co_3_O_4_/Ti cathode. Water Res..

[14] Garcia-Segura S., Ocon J.D., Chong M.N. (2018). Electrochemical oxidation remediation of real wastewater effluents—A review. Process Saf. Environ..

[15] Yu H., Li Y., Zhao M., Dong H., Yu H.B., Zhan S.H., Zhang L. (2015). Energy-saving removal of methyl orange in high salinity wastewater by electrochemical oxidation via a novae Ti/SnO_2_-Sb anode-Air diffusion cathode system. Catal. Today.

[16] Qiao J., Xiong Y.Z. (2021). Electrochemical oxidation technology: A review of its application in high-efficiency treatment of wastewater containing persistent organic pollutants. J. Water Process Eng..

[17] Ganzoury M.A., Ghasemian S., Zhang N., Yagar M., de Lannoy C.F. (2022). Mixed metal oxide anodes used for the electrochemical degradation of a real mixed industrial wastewater. Chemosphere.

[18] Markou V., Kontogianni M.C., Frontistis Z., Tekerlekopoulou A.G., Katsaounis A., Vayenas D. (2017). Electrochemical treatment of biologically pre-treated dairy wastewater using dimensionally stable anodes. J. Environ. Manag..

[19] Zhou Y., Zhao K., Hu C.Z., Liu H.J., Wang Y., Qu J.H. (2018). Electrochemical oxidation of ammonia accompanied with electricity generation based on reverse electrodialysis. Electrochim. Acta.

[20] Zöllig H., Fritzsche C., Morgenroth E., Udert K.M. (2015). Direct electrochemical oxidation of ammonia on graphite as a treatment option for stored source-separated urine. Water Res..

[21] Ghazouani M., Akrout H., Bousselmi L. (2017). Nitrate and carbon matter removals from real effluents using Si/BDD electrode. Environ. Sci. Pollut. Res..

[22] Couto A.B., Oishi S.S., Ferreira N.G. (2016). Enhancement of nitrate electroreduction using BDD anode and metal modified carbon fiber cathode. J. Ind. Eng. Chem..

[23] Yao J.C., Pan B.J., Shen R.X., Yuan T.B., Wang J.D. (2019). Differential control of anode/cathode potentials of paired electrolysis for simultaneous removal of chemical oxygen demand and total nitrogen. Sci. Total Environ..

[24] Mook W.T., Chakrabarti M.H., Aroua M.K., Khan G.M.A., Ali B.S., Islam M.S., Abu Hassan M.A. (2012). Removal of total ammonia nitrogen (TAN), nitrate and total organic carbon (TOC) from aquaculture wastewater using electrochemical technology: A review. Desalination.

[25] Yao J.C., Mei Y., Yuan T.B., Chen J., Pan H., Wang J.D. (2021). Electrochemical removal of nitrate from wastewater with a Ti cathode and Pt anode for high efficiency and N_2_ selectivity. J. Electroanal. Chem..

[26] Cirmi D., Aydin R., Koleli F. (2015). The electrochemical reduction of nitrate ion on polypyrrole coated copper electrode. J. Electroanal. Chem..

[27] Shih Y.J., Huang Y.H., Huang C.P. (2018). Electrocatalytic ammonia oxidation over a nickel foam electrode: Role of Ni(OH)_2(s)_-NiOOH(s) nanocatalysts. Electrochim. Acta.

[28] Li M., Feng C.P., Zhang Z.Y., Yang S.J., Sugiura N. (2010). Treatment of nitrate contaminated water using an electrochemical method. Bioresour. Technol..

[29] Li W., Xiao C.W., Zhao Y., Zhao Q.Q., Fan R., Xue J.J. (2016). Electrochemical reduction of high-concentrated nitrate using Ti/TiO_2_ nanotube array anode and Fe cathode in dual-chamber cell. Catal. Lett..

[30] Mandal P., Dubry B.K., Gupta A.K. (2017). Review on landfill leachate treatment by electrochemical oxidation: Drawbacks, challenges and future scope. Waste Manag..

[31] Zou J.X., Peng X.L., Li M., Xiong Y., Wang B., Dong F.Q., Wang B. (2017). Electrochemical oxidation of COD from real textile wastewaters: Kinetic study and energy consumption. Chemosphere.

[32] Chen J.M., Xia Y.J., Dai Q.Z. (2015). Electrochemical degradation of chloramphenicol with a novel Al doped PbO_2_ electrode: Performance, kinetics and degradation mechanism. Electrochim. Acta.

[33] Yao J.C., Chen A.N., Ye R.H., Wang J.D., Pan H., Xu D.M., Chen J., Mei Y., Hrynsphan D., Savitskaya T. (2021). Stepping control of electrochemical process for simultaneous removal of COD and ammonia with high efficiency and energy saving. J. Electrochem. Soc..

[34] Chen G.H. (2004). Electrochemcial technologies in wastewater treatment. Sep. Purif. Technol..

[35] Yao J.C., Zhou M.M., Wen D.N., Xue Q.W., Wang J.D. (2016). Electrochemical conversion of ammonia to nitrogen in non-chlorinated aqueous solution by controlling pH value. J. Electroanal. Chem..

[36] Zhu R.Y., Yang C.Y., Zhou M.M., Wang J.D. (2015). Industrial park wastewater deeply treated and reused by a novel electrochemical oxidation reactor. Chem. Eng. J..

[37] Li M., Feng C.P., Zhang Z.Y., Sugiura N. (2009). Efficient electrochemical reduction of nitrate to nitrogen using Ti/IrO_2_-Pt anode and different cathodes. Electrochim. Acta.

[38] Dia O., Drogui P., Buelna G., Dubé R. (2017). Strategical approach to prevent ammonia formation during electrocoagulation of landfill leachate obtained from a biofiltration process. Sep. Purif. Technol..

[39] Reyter D., Bélanger D., Roué L. (2011). Optimization of the cathode material for nitrate removal by a paired electrolysis process. J. Hazard. Mater..

[40] Griffiths P.R., de Haseth J.A. (1986). Fourier Transform Infrared Spectrometry.

[41] Kapałka A., Cally A., Neodo S., Comninellis C., Wächter M., Udert K.M. (2010). Electrochemical behavior of ammonia at Ni/Ni(OH)_2_ electrode. Electrochem. Commun..

[42] Anglada A., Urtiaga A., Ortiz I. (2009). Pilot scale performance of the electro-oxidation of landfill leachate at boron-doped diamond anodes. Environ. Sci. Technol..

[43] Yang J.B., Du C.W., Shen Y.Z., Jian J.M. (2013). Rapid determination of nitrate in Chinese cabbage using fourier transforms mid-infrared spectroscopy. Chin. J. Anal. Chem..

[44] Valverde J.L., de Lucas A., Dorado F., Romero A., Garcia P.B. (2005). Study by in situ FTIR of the SCR of NO by propene on Cu^2+^ ion-exchanged Ti-PILC. J. Mol. Catal. A-Chem..

[45] Talhi B., Monette F., Azzouz A. (2011). Effective and selective nitrate electroreduction into nitrogen through synergistic parameter interactions. Electrochim. Acta.

[46] Zhao J., Zhu C.Z., Lu J., Hu C.J., Peng S.C., Chen T.H. (2014). Electro-catalytic degradation of bisphenol A with modified Co_3_O_4_/β-PbO_2_/Ti electrode. Electrochim. Acta.

[47] Wang Y., Qu J.H., Wu R.C., Lei P.J. (2006). The electrocatalytic reduction of nitrate in water on Pd/Sn-modified activated carbon fiber electrode. Water Res..

[48] Lacasa E., Cañizares P., Llanos J., Rodrigo M.A. (2012). Effect of the cathode material on the removal of nitrates by electrolysis in non-chloride media. J. Hazard. Mater..

[49] Cai Q.Q., Wu M.Y., Li R., Deng S.H., Lee B.C.Y., Ong S.L., Hu J.Y. (2020). Potential of combined advanced oxidation-Biological process for cost-effective organic matters removal in reverse osmosis concentrate produced from industrial wastewater reclamation: Screening of AOP pre-treatment technologies. Chem. Eng. J..

[50] Bustillo-Lecompte C.F., Mehrvar M. (2016). Treatment of an actual slaughterhouse wastewater by integration of biological and advanced oxidation processes: Modeling, optimization, and cost-effectiveness analysis. J. Environ. Manag..

