# Supplementary material for: Process Optimization of Electrochemical Treatment of COD and Total Nitrogen Containing Wastewater
Source: Int J Environ Res Public Health. 2022 Jan 13;19(2):850. doi: 10.3390/ijerph19020850 (PMC8776051; doi:10.3390/ijerph19020850)
Supplement: Supplementary file 1 [file ijerph-19-00850-s001.zip › ijerph-1479904-supplementary.pdf]

International Journal of
Environmental Research and Public Health

MDPI

Supporting information for

Process Optimization of Electrochemical Treatment of COD and Total Nitrogen Containing Wastewater

Jiachao Yao [1], Yu Mei [1], Junhui Jiang [2], Guanghua Xia [3] and Jun Chen [1,*]

[1] College of Biology and Environmental Engineering, Zhejiang Shuren University, Hangzhou 310015, China; jcyao@zjut.edu.cn (J.Y.); imy1220@zjut.edu.cn (Y.M.)
[2] The Engineering Technology Center of Pollution Control in Taizhou, Taizhou 318000, China; 14060601003@pop.zjgsu.edu.cn
[3] College of Life Science, Taizhou University, Taizhou 318000, China; zhouwuluyao@sjtu.edu.cn
* Correspondence: bec@zjut.edu.cn; Tel.: +86-571-88320448

Table S1. The efficiencies for COD and TN removal at different initial pH values, Cl^- concentrations and current densities.

Parameters	Conditions	COD removal efficiency (%)	−100 × K (min^{-1})	R^2	TN removal efficiency (%)	−100 × K' (mg L^{-1} min^{-1})	R^2
Initial pH value	3	80.4	1.393	0.997	16.0	14.073	0.995
	6	77.4	1.307	0.996	18.1	16.434	0.997
	7	75.8	1.207	0.988	11.6	9.443	0.982
	9	72.7	1.067	0.990	17.6	15.994	0.995
	11	63.9	0.870	0.996	19.2	16.985	0.996
Cl^- concentration (mg L^{-1})	0	77.4	1.307	0.996	18.1	16.434	0.998
	250	88.6	1.881	0.988	26.0	22.465	0.992
	500	90.8	2.017	0.988	46.7	39.990	0.999
	1000	92.7	2.198	0.998	82.0	69.324	0.996
	1500	94.8	2.420	0.987	87.0	103.694	0.999
Current density (mA cm^{-2})	5.0	78.4	1.269	0.997	72.6	60.324	0.983
	7.5	85.7	1.678	0.998	75.8	64.776	0.987
	10.0	92.7	2.198	0.998	82.0	68.994	0.990
	12.5	95.3	2.471	0.997	86.0	79.181	0.997

Table S2. Selected results reported on energy consumption of actual wastewater treatment by electrochemical method.

Electrode material		Experimental conditions	Results		Ref.
Anode	Cathode		Removal efficiency	Energy consumption (kWh m^{-3})	
Fe	Fe	7.8 mA cm^{-2}; pH 5.5; 120 min electrolysis	COD: 62%	42	(Sahu et al., 2015)
BDD	-	470 mg L^{-1} COD; 8 mA cm^{-2}; 180 min electrolysis	COD: 80%	28.6	(Tsantaki et al., 2012)
Graphite	Graphite	anode potential of 1.31 V (vs. SHE); 25 °C; pH 9	COD: 77%<br>NH_4^+: 38%	24.7	(Zöllig et al., 2015)
Ti/IrO_2-RuO_2-TiO_2	Ti	10 mA cm^{-2}; pH 2; 2 g L^{-1} Cl^-; flow rate of 50 mL min^{-1}	COD: 70%	23.2	(Aravind et al., 2018)
Ti/PbO_2	Stainless steel	780 mg L^{-1} COD; flow rate of 420 L h^{-1}; pH 8.2; current of 2 A; 3 h electrolysis	COD: >79%	90	(Panizza et al., 2010)
Ti/IrO_2	Ni	620 mg L^{-1} NO_3^-; cathode potential of −1.1 V (vs. SCE); 180 min electrolysis	NO_3^-: 13%	17.7	(Reyter et al., 2011)
Ti/PbO_2	Ti	337.57 mg L^{-1} COD; 120.53 mg-N L^{-1} TN; 10 mA cm^{-2}; flow rate of 300 mL min^{-1}; 120 min electrolysis	COD: 79.1%<br>TN: 87.0%	13.3	In this study

1. O.P. Sahu, P.K. Chaudhari, Electrochemical treatment of sugar industry wastewater: COD and color removal, J. Electroanal. Chem. 739 (2015) 122-129.
2. E. Tsantaki, T.Velegraki, A. Katsaounis, D. Mantzavinos, Anodic oxidation of textile dyehouse effluents on boron-doped diamond electrode. J. Hazard. Mater. 207-208 (2012) 91-96.
3. H. Zöllig, C. Fritzsche, E. Morgenroth, K.M. Udert, Direct electrochemical oxidation of ammonia on graphite as a treatment option for stored source-separated urine. Water Res. 69 (2015) 284–294.
4. P. Aravind, H. Selvaraj, S. Ferro, G.M. Neelavannan, M. Sundaram, A one-pot approach: Oxychloride radicals enhanced electrochemical oxidation for the treatment of textile dye wastewater trailed by mixed salts recycling. J. Clean. Prod. 182 (2018) 246-258.
5. M. Panizza, M. Delucchi, I. Sirés, Electrochemical process for the treatment of landfill leachate, J. Appl. Electrochem. 40 (2010) 1721-1727.
6. D. Reyter, D. Bélanger, L. Roué, Optimization of the cathode material for nitrate removal by a paired electrolysis process. J. Hazard. Mater. 192 (2011) 507-513.

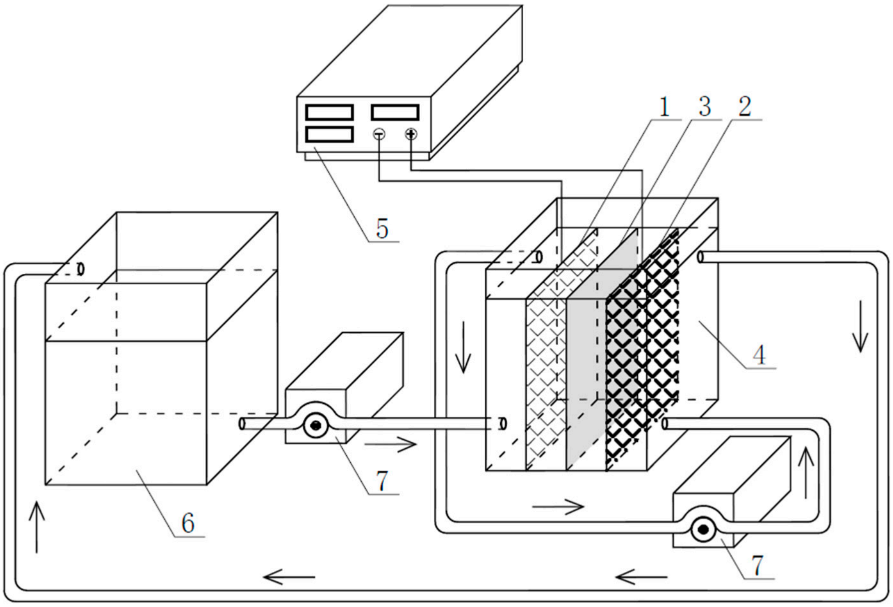

Figure S1. Schematic diagram of the divided electrolysis cell. 1. cathode; 2. anode; 3. proton-exchange membrane; 4. reaction cell; 5. power supply; 6. reservoir; 7. peristaltic pump.

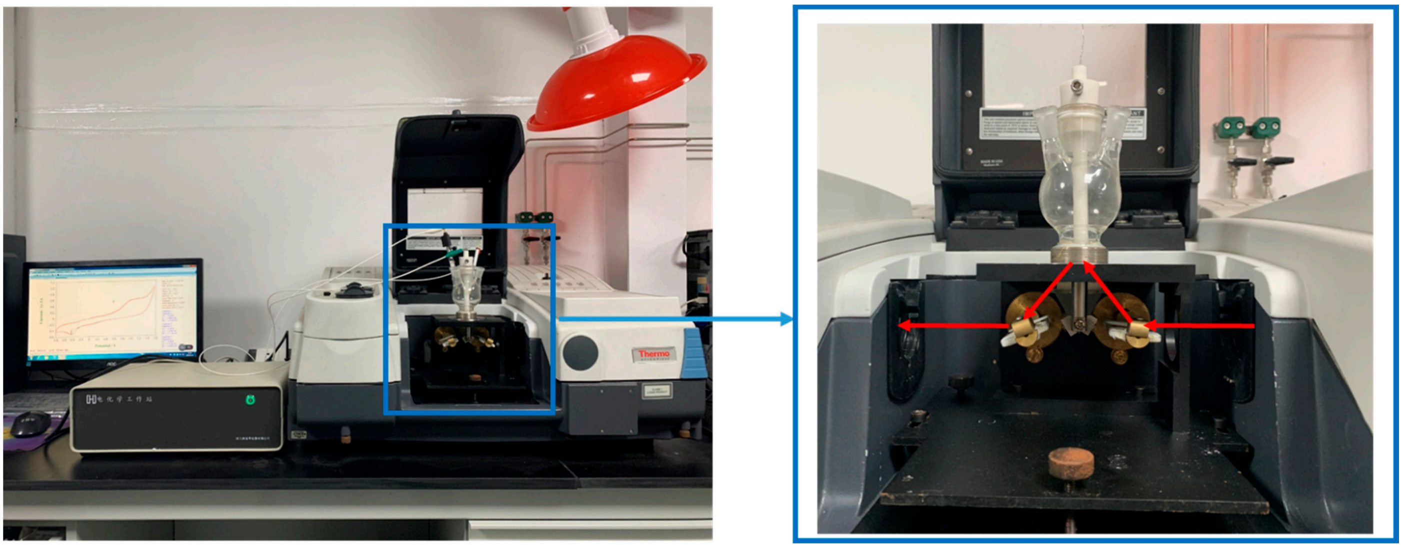

Figure S2. Schematic diagram of in-situ FTIR.

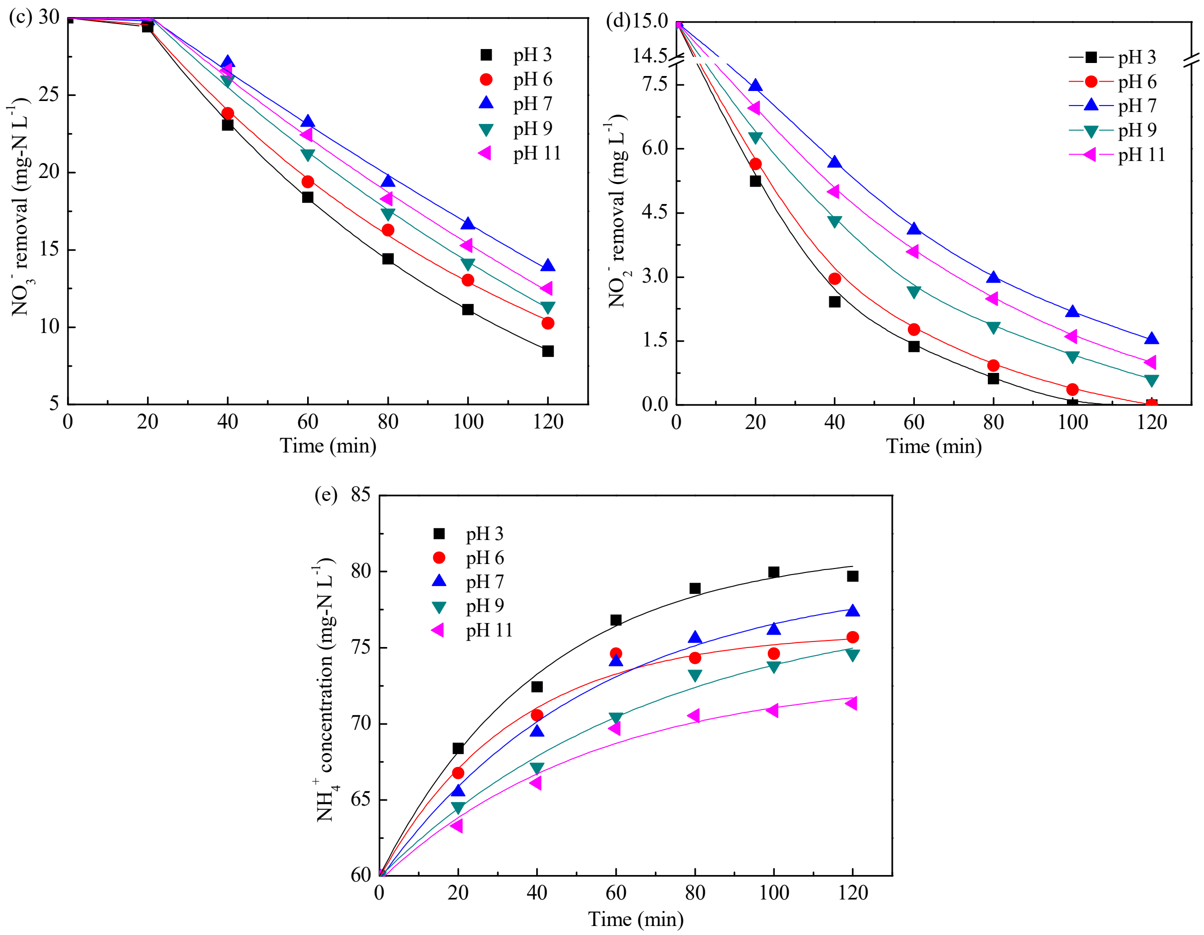

Figure S3. Influence of initial pH on NO_3^- (c), NO_2^- (d), and NH_4^+ (e) variations. Diluted H_2SO_4 and NaOH solutions were used for pH adjustments, and pH 6 was the natural pH of simulated wastewater.
